# Development of a Bioluminescent Imaging Mouse Model for SARS-CoV-2 Infection Based on a Pseudovirus System

**DOI:** 10.3390/vaccines11071133

**Published:** 2023-06-22

**Authors:** Xi Wu, Nana Fang, Ziteng Liang, Jianhui Nie, Sen Lang, Changfa Fan, Chunnan Liang, Weijin Huang, Youchun Wang

**Affiliations:** 1 Division of HIV/AIDS and Sex-Transmitted Virus Vaccines, Institute for Biological Product Control, National Institutes for Food and Drug Control (NIFDC), Beijing 102629, China; wuxi@nifdc.org.cn (X.W.); huangweijin@nifdc.org.cn (W.H.); 2 National Vaccine and Serum Institute, Beijing 101111, China; m18210828042@163.com; 3 Chinese Academy of Medical Sciences & Peking Union Medical College, Beijing 100006, China; 4 National Rodent Laboratory Animal Resources Center, Institute for Laboratory Animal Resources, National Institutes for Food and Drug Control (NIFDC), Beijing 102629, China; 5 Institute of Medical Biology, Chinese Academy of Medical Sciences, Kunming 650118, China

**Keywords:** SARS-CoV-2 pseudovirus, mouse model, in vivo bioluminescent

## Abstract

Coronavirus disease 2019 (COVID-19), caused by severe acute respiratory syndrome coronavirus 2 (SARS-CoV-2), remains widely pandemic around the world. Animal models that are sensitive to the virus are therefore urgently needed to evaluate potential vaccines and antiviral agents; however, SARS-CoV-2 requires biosafety level 3 containment. To overcome this, we developed an animal model using the intranasal administration of SARS-CoV-2 pseudovirus. As the pseudovirus contains the firefly luciferase reporter gene, infected tissues and the viral load could be monitored by in vivo bioluminescent imaging. We used the model to evaluate the protective efficacy of monoclonal antibodies and the tissue tropism of different variants. The model may also be a useful tool for the safe and convenient preliminary evaluation of the protective efficacy of vaccine candidates against SARS-CoV-2, as well as the treatment efficacy of anti-viral drugs.

## 1. Introduction

By March 2023, over 675 million confirmed cases of coronavirus disease 2019 (COVID-19), including over 6.87 million deaths, had been reported to the World Health Organization. The combination of high transmissibility and virulence of severe acute respiratory syndrome coronavirus 2 (SARS-CoV-2), the causative agent of COVID-19, underscores the urgent need to develop highly effective vaccines and antiviral agents, which can provide protection against rapidly evolving variants.

Both in vitro and in vivo methods have been developed to evaluate the efficacy of antiviral agents, among which mice are the most frequently used animal model. Mice offer the advantages of a low cost, high biological similarity to humans and a short lifecycle. However, mice cannot be infected by SARS-CoV-2 because of amino acid differences between mouse ACE2 (mACE2) and human ACE2 (hACE2) [1]. Multiple strategies have been applied to sensitize mice to SARS-CoV-2 infection, including expressing hACE2 via transgenic, knock-in (KI), and viral-vector transduction methods [2,3,4,5]. Other strategies include modifying the virus to adapt it to mice [5]. Among these models, a hACE2 KI mouse model was developed by replacing mACE2 with hACE2 cDNA. The hACE2 KI mice were infected intranasally and high viral loads were observed in the respiratory system, including the lungs and trachea [2]. However, research with SARS-CoV-2 must be performed in a biosafety level 3 (BSL-3) laboratory, which has hampered the development of antiviral therapeutic agents. Recently, pseudovirus-based neutralization assays have been established, which show a good correlation with human virus infection. Application of the pseudovirus allows for the evaluation of anti-SARS-CoV-2 agents in laboratories with a lower level of containment (BSL-2) [6,7].

Here, to facilitate the investigation of antiviral agents, we established a SARS-CoV-2 pseudovirus infection mouse model. Using pseudovirus harboring the luciferase gene, we optimized an in vivo bioluminescent model to mimic natural infection with SARS-CoV-2, which can be continuously monitored in live animals. The protection efficiencies of monoclonal neutralizing antibodies were evaluated and the infection efficiencies of different variants were detected using this model.

## 2. Materials and Methods

### 2.1. Cells

HEK293T (American Type Culture Collection (ATCC), CRL-3216) and Vero E6 (ATCC, CRL-1586) were cultured in high-glucose Dulbecco’s modified Eagle’s medium (DMEM; HyClone, Logan, UT, USA) supplemented with 10% fetal bovine serum (FBS; Gibco, Billings, MT, USA) and 1% penicillin–streptomycin (Gibco) in a humidified 5% CO_2_ atmosphere at 37 °C.

### 2.2. Production of Single Cycle (sc) Vesicular Stomatitis Virus (VSV)-SARS-CoV-2

The Spike gene of SARS-CoV-2 was codon-optimized for human cells and expressed on plasmid pcDNA3.1. Plasmid expressing spike of the indicated variant was then transfected into 293T cells using Lipofectamine 3000 reagent (Invitrogen, L3000015, Carlsbad, CA, USA) following the manufacturer’s instructions. At the same time, the transfected cells were infected with G gene-deleted (G*ΔG) VSV, which harbored a luciferase reporter gene, at a multiplicity of infection (MOI) of 4. Six hours after infection, the supernatants were removed. Cells were washed with 2% FBS/PBS three times, then fresh DMEM with 10% FBS was added. At 24 h post-infection, the supernatants containing scVSV-SARS-CoV2 were harvested, filtered through a 0.45 nm filter, and stored at −70 °C until use.

### 2.3. Generation of rVSV-SARS-CoV-2

rVSV-SARS-CoV-2 was rescued from cDNA clones. Briefly, a plasmid encoding the T7 promoter for the VSV antigenome modified by a replacement of the G gene with a 21-amino acid (aa)-deleted codon-optimized SARS-CoV-2 spike gene with the indicated mutations and a luciferase reporter gene was inserted into the genome as a separate transcriptional unit before the spike gene was constructed. Plasmid-based rescue of the replication-competent VSV-based pseudovirus was carried out as described previously [8]. Briefly, BHK21 cells were infected with vaccinia virus vTF7-3, which expresses T7 polymerase, for 2 h. Then, the supernatant was discarded and cells were transfected with plasmid encoding the VSV antigenome, along with helper plasmids encoding the T7 promoter for the VSV N, P, G, and L genes using Lipofectamine 3000 reagent. Cells were cultured for an additional 48 h and the supernatants were collected and filtered through a 0.22 nm filter and passaged in Vero E6 cells. The virus was further passaged and amplified every 2–3 days, three to four times. The viral RNA was then extracted and reverse transcription PCR was performed. The full-length spike gene was amplified and sequenced. The supernatant containing the virus was aliquoted and stored at −80 °C.

### 2.4. Animal Experiments

The study was approved by the Animal Care and Use Committee at the National Institute for Food and Drug Control (NIFDC). Three-week-old C57BL/6 and hACE2 KI mice were obtained from the Institute for Laboratory Animal Resources, NIFDC. The mice were infected with scVSV-SARS-CoV-2 or rVSV-SARS-CoV-2 harboring spike protein with the indicated mutation via intraperitoneal (IP), intravenous (IV), intragastric (IG), or intranasal (IN) injection. Two to four mice were used in each group. Three independent experiments were conducted and representative images are shown. The bioluminescent signals were monitored at the indicated timepoints post-infection.

### 2.5. Bioluminescent Imaging (BLI) Analysis

Mice were subjected to IP injection of the substrate, d-luciferin (50 mg/kg body weight), and were then anaesthetized with isoflurane at 10 min post-injection. In vivo bioluminescence was monitored using the IVIS Lumina Series III Imaging System. Images were acquired with an exposure time of 60 s. The images were displayed in color according to pseudotype, ranging from red to blue.

### 2.6. Viral Load Assay

Quantification of the virus was performed using real-time PCR. RNA was extracted from the lungs of control and monoclonal antibody (mAb)-treated mice. Reverse transcription was performed using the reverse transcription PCR kit (Thermo Fisher Scientific, Waltham, MA, USA). SYBR green quantitative real-time PCR was performed using a LightCycler 480 Real-Time PCR System (Roche). VSV-P mRNA was quantified. The sequences of primers used for this analysis were as follows: forward: 5′-GCAATTGACAGCTCTTCTGCTCA-3′, and reverse: 5′-GTCGTCAATCCTCCGGTACTATCATCTG-3′.

### 2.7. Statistical Analysis

All data were analyzed and graphs were generated using GraphPad Prism 6.0 software (GraphPad, San Diego, CA, USA). *p*-values of <0.05 were considered statistically significant.

## 3. Results

### 3.1. Packing Optimization and Generation of Single Cycle and Replication-Competent VSV-SARS-CoV-2 Pseudovirus

**Figure 1:**
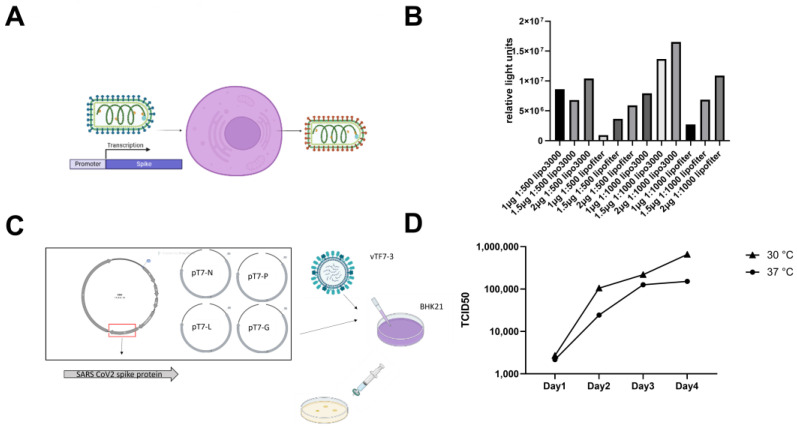
Packing optimization and rescue of scVSV-SARS-CoV-2 and rVSV-SARS-CoV-2. (**A**) A scheme for scVSV preparation. SARS-CoV-2 spike protein with a 21-amino acid deletion at the C-terminus was pseudotyped on the surface of VSV particles that carry the Fluc reporter gene. (**B**) Optimization of the ratio of spike expression vectors, G*ΔG-VSV, and transfection reagents. (**C**) A scheme for rVSV-SARS-CoV-2 rescue. BHK21 cells were infected with vaccinia virus vTF7-3, which expresses T7 polymerase for 2 h, then transfected with T7 promoter-driven VSV N, P, L, and G genes and the antigenome of VSV harboring a luciferase reporter. After 48 h of culture, the supernatants were collected and filtered through a 0.22 μm filter. Then, rVSV-SARS-CoV-2 was further amplified by passage in Vero cells. (**D**) The bioluminescence signals were monitored after infecting cells with rVSV-SARS-CoV-2 at the indicated timepoints and temperature.

To generate scVSV-SARS-CoV-2 pseudovirus, 293T cells were transfected with a plasmid expressing SARS-CoV-2 spike protein with the D614G mutation (S-D614G) and infected with G gene-deleted VSV harboring the firefly luciferase (Fluc) reporter, simultaneously (Figure 1A). To obtain a high titer of pseudotyped SARS-CoV-2 virus, the amount of spike expression vector, and the ratio of G*ΔG-VSV and transfection reagent were optimized. We observed that cells in the six-well plate transfected with 2 µg of spike-expressing vectors by Lipofectamine 3000 and infected with G*ΔG-VSV at a ratio of 1:1000, producing the highest titer of scVSV-SARS-CoV-2 (Figure 1B).

To generate a replication-competent VSV-SARS-CoV-2 (rVSV-SARS-CoV-2) pseudovirus, we synthetized a plasmid harboring the VSV antigenome with a luciferase reporter inserted between VSV-M and VSV-G. Then, the G gene was replaced by S-D614G with a 21-aa deletion at the C-terminus. This 21-aa sequence has been reported to be essential for the efficient replication of rVSV-SARS-CoV-2. Plasmid-based rescue of rVSV-SARS-CoV-2 was carried out as described previously [8] (Figure 1C) and generated a slowly replicating virus bearing the spike protein in the envelope. This virus was then amplified by passage in Vero E6 cells. Virus replication dynamics were characterized after low MOI (0.1) infection at 30 °C and 37 °C, respectively. We observed high viral titers 72–96 h following infection, and the viral titers were significantly higher at 30 °C (Figure 1D).

### 3.2. Construction and Optimization of a Bioluminescent Imaging Mouse Model

**Figure 2:**
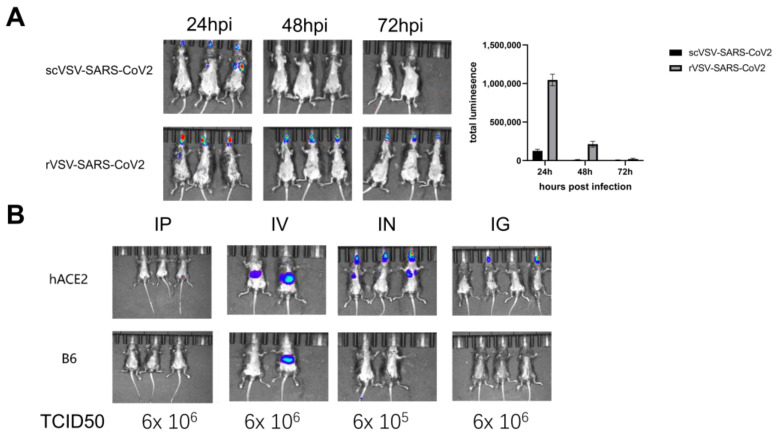
Construction and optimization of a bioluminescent imaging mouse model. (**A**) Comparison between scVSV-SARS-CoV-2 and rVSV-SARS-CoV-2 infected hACE2 KI mice. Mice were inoculated with the indicated pseudovirus harboring S-D614G via intranasal injection (2 × 10^5^ TCID_50_/mouse). The virus distribution and dissemination were monitored by bioluminescent imaging (BLI) on days 1, 2, and 3 post-infection (left panel) and quantified (right panel). (**B**) Comparison of different infection routes (IP, IV, IN, and IG) in hACE2 KI mice and C57BL/6 mice. Mice were inoculated with rVSV-SARS-CoV-2 harboring S-D614G at the indicated TCID_50_. The virus distribution and infection intensity were monitored by the bioluminescent signal at 24 h post-infection.

To establish a robust, bioluminescent imaging SARS-CoV-2 mouse model, we first tested the infection dynamics of scVSV-SARS-CoV-2 and rVSV-SARS-CoV-2 in the mouse model. hACE2 KI mice were infected with single cycle or replication-competent VSV-SARS-CoV-2 pseudovirus, harboring S-D614G, intranasally at a dose of 2 × 10^5^ TCID_50_. Bioluminescence signals were monitored at 24, 48, and 72 h post-infection (Figure 2A). The signal intensity peaked at 24 h post-infection, then gradually decreased. Replication-competent pseudovirus-infected mice showed significantly higher signals than the single cycle virus, so replication-competent pseudovirus was selected for further infection studies and 24 h post-infection was selected as the optimal timepoint to carry out the assays. To further evaluate specificity and infection ability, different infection routes, including intraperitoneal (IP), intravenous (IV), intragastric (IG), and intranasal (IN) injection, were compared in hACE2 KI mice and C57BL/6 mice. To achieve an intensive infection process, mice were administrated with a maximum volume (1 mL through the IV, IP, and IG routes, 100 μL through the IN route) of virus at 6 × 10^6^ TCID_50_/mL concentration (Figure 2B). Bioluminescence signals were detected in the IV and IN groups, but not in the IP and IG groups. C57BL/6 mice also showed an enrichment of bioluminescence signals in the liver in the IV group, suggesting nonspecific infection through this route. By contrast, virus infected though the IN route was enriched in the nose and lungs in hACE2 KI mice, while signals were undetectable in C57BL/6 mice. These data showed that the dissemination of bioluminescence signals in the IN group were stable and more specific than in mice in the other groups.

### 3.3. Evaluation of Monoclonal Neutralizing Antibodies Protection in the Mouse Model

**Figure 3:**
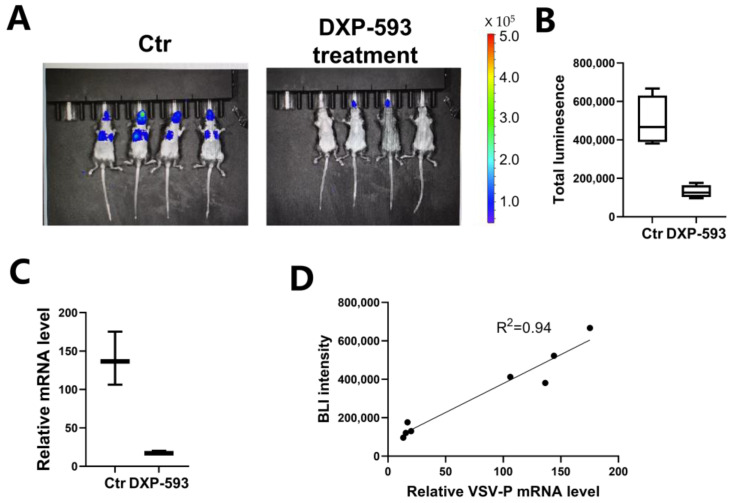
Evaluation of monoclonal neutralizing antibody protection efficiency in the mouse model. (**A**) Anti-SARS-CoV-2 mAbs were injected to evaluate their preventive efficacies. The mAbs were injected 2 days and 1 day before the inoculation of rVSV-SARS-CoV-2 to determine their preventive efficacies. Flux was measured at 1 day post-infection. (**B**) Quantification of the total flux for each mouse. (**C**) Quantification of VSV in the lungs of control and mAb-treated mice was performed by RT-PCR of VSV-P mRNA levels and were normalized to GAPDH levels. Values are shown as the means *±* SEMs of three independent experiments. (**D**) Linear regression between the relative VSV-P mRNA levels in the lungs and the total bioluminescence of the lungs.

With the continuation of the COVID-19 pandemic around the world, mouse models to assess potential antiviral agents are urgently needed. BGB-DXP593 is a monoclonal neutralizing antibody identified from the B cells of convalescent COVID-19 patients [9] that can neutralize pseudovirus harboring S-D614G in vitro [10]. We used our infection model to assess the in vivo prevention efficacies of this antibody. Mice were administered two doses of the BGB-DXP593 mAb individually (20 mg/kg) via the intravenous (IV) route before pseudovirus exposure (days −2 and −1) and were then challenged with IN administration of rVSV-SARS-CoV-2 (day 0). Nearly complete protection was conferred by BGB-DXP593 mAb (Figure 3A,B). Quantitative PCR was performed using primers targeting the VSV-P gene, which resides in the backbone of the pseudovirus, and the data were normalized to that of the *Gapdh* gene (Figure 3C). The results confirmed that the viral load significantly decreased in the mAb-treated group. We further performed linear regression and found a good correlation between the relative VSV-P mRNA level and BLI (r^2^ = 0.94), suggesting that bioluminescence intensity reflects the viral load in vivo (Figure 3D).

### 3.4. Evaluation of Tissue Tropism of Different Variants in the Mouse Model

**Figure 4:**
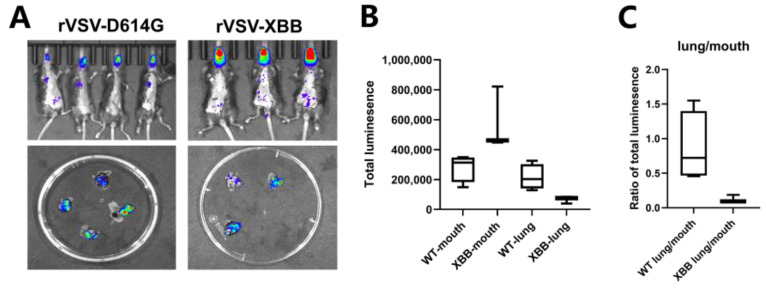
Evaluation of tissue tropism of different variants (**A**) Mice were inoculated with rVSV-SARS-CoV-2 harboring the indicated S variants by intranasal injection (2 × 10^5^ TCID_50_/mouse). Flux was measured at 1 day post-infection. Lungs were dissected for BLI. (**B**) Quantification of the total flux intensity in the mouths and lungs of each group. (**C**) The ratio of flux in the lung region to flux in the mouth region was determined for each mouse.

Because of the highly variant nature of SARS-CoV-2, evaluation of the pathogenesis and virulence of different variants is important. Pseudoviruses enable different mutants to be easily constructed. We constructed rVSV-SARS-CoV-2 harboring the S gene from XBB strain. rVSV-SARS-CoV-2 (D614G) and rVSV-SARS-CoV-2 (XBB) reached similar titers on Vero cells and particle numbers were quantified by quantitative PCR. We then tested their infection ability in vivo. Mice infected with rVSV-SARS-CoV-2 (D614G) showed viral loads in the mouth and/or lungs (Figure 4A,B), while mice infected with rVSV-SARS-CoV-2 (XBB) showed significantly lower viral loads in the lungs (Figure 4C).

## 4. Discussion

The high transmissibility and virulence of SARS-CoV-2 dictate the need for BSL-3 containment, which presents a major obstacle to research. The objective of this study was to establish a safe and convenient model mimicking SARS-CoV-2 infection. A typical pseudovirus comprises the genomic backbone from one virus and the envelope protein from another virus. The genomic backbone is often modified and reporter genes may be added, which limits the replication and/or reduces the virulence of the virus. Pseudoviruses thereby allow researchers to safely work with a virus under BSL-2 containment. In this study, we used a VSV-based pseudovirus that could produce a high titer of SARS-CoV-2. Different spike variants of SARS-CoV-2 can be easily incorporated into the pseudovirus and studied without the need for live viruses.

In a previous study, lentivirus-based SARS-CoV-2 pseudoviruses were used to model SARS-CoV-2 infection following transduction with adenoviruses expressing hACE2 [11]. However, because of the replication-incompetent nature of the pseudovirus and inefficient transduction of hACE2 by adenovirus, the infection of SARS-CoV-2 pseudovirus was limited to the upper respiratory tract. We compared the infection efficiency of scVSV-SARS-CoV-2 and rVSV-SARS-CoV-2, and found the bioluminescence signal to be significantly higher in rVSV-SARS-CoV-2-infected mice than scVSV-SARS-CoV-2-infected mice, indicating that replication occurred in vivo. Furthermore, the rVSV-SARS-CoV-2 infection model exhibited lung infection, which appeared to mimic human SARS-CoV-2 infection. However, we noticed that the intensity of the bioluminescence signal was highest at 24 h post-infection, then gradually decreased, indicating that rVSV-SARS-CoV-2 did not continuously replicate in mice. The generation of hACE2 in an immunodeficient background may be useful to study the continuous infection process and the tissue distribution of rVSV-SARS-CoV-2.

It was previously reported that the SARS-CoV-2 variant Omicron BA.1 was associated with a lower risk of hospitalization and severe illness compared with the Delta variant [12]. Consistent with this observation, when hACE2 mice were infected with rVSV-SARS-CoV-2 harboring the spike protein of the XBB variant, the viral load in the lungs was much lower compared with those infected with wild-type rVSV-SARS-CoV-2. Some mutations have been suggested to influence cell surface entry pathways, which may affect tissue tropism. Further studies are needed to determine the crucial mutation site(s) responsible for the tissue tropism of different SARS-CoV-2 variants.

With the rapid evolution of SARS-CoV-2, the investigation and verification of new antibodies is urgently needed. We used a mouse model to evaluate the protective efficacy of mAbs administered via the IV route. Our model was effective in evaluating broad-acting neutralizing antibodies via the infection of pseudoviruses with different envelope proteins. Our model could be further expanded to evaluate the protective efficacy of vaccines [13,14] and to screen for anti-viral reagents targeting virus entry and fusion with the cell membrane.

In summary, we established an in vivo infection model using a replication-competent pseudovirus, which will facilitate the evaluation of vaccines, antibodies, and anti-viral reagents against SARS-CoV-2, and provide a safer and more flexible alternative to the use of live virus.

## Data Availability

The data presented in this study are available on request from the corresponding author. The data are not publicly available due to privacy.
